# Yeast AP-1 like transcription factors (Yap) and stress response: a current overview

**DOI:** 10.15698/mic2019.06.679

**Published:** 2019-05-28

**Authors:** Claudina Rodrigues-Pousada, Frédéric Devaux, Soraia M. Caetano, Catarina Pimentel, Sofia da Silva, Ana Carolina Cordeiro, Catarina Amaral

**Affiliations:** 1 Instituto de Tecnologia Química e Biológica Anónio Xavier, Universidade Nova de Lisboa, Avenida da República, EAN, Oeiras 2781-901, Oeiras, Portugal.; 2 Sorbonne Université, CNRS, Institut de Biologie Paris-Seine, Laboratory of Computational and Quantitative Biology, F-75005, Paris, France.

**Keywords:** yeast, Yap factors, cis-elements, bZIP, stress

## Abstract

Yeast adaptation to stress has been extensively studied. It involves large reprogramming of genome expression operated by many, more or less specific, transcription factors. Here, we review our current knowledge on the function of the eight Yap transcription factors (Yap1 to Yap8) in *Saccharomyces cerevisiae*, which were shown to be involved in various stress responses. More precisely, Yap1 is activated under oxidative stress, Yap2/Cad1 under cadmium, Yap4/Cin5 and Yap6 under osmotic shock, Yap5 under iron overload and Yap8/Arr1 by arsenic compounds. Yap3 and Yap7 seem to be involved in hydroquinone and nitrosative stresses, respectively. The data presented in this article illustrate how much knowledge on the function of these Yap transcription factors is advanced. The evolution of the Yap family and its roles in various pathogenic and non-pathogenic fungal species is discussed in the last section.

## INTRODUCTION

The yeast *Saccharomyces cerevisiae* has been used in research for more than one hundred years, and it is generally regarded as the most well understood eukaryotic organism in the stress response field. The sensing and transduction of the stress signals into different cellular compartments induce a genetic reprogramming, which leads to a transient arrest of normal cellular processes, with a decrease in the expression of housekeeping genes and protein synthesis. In addition, there is an induction of the expression of genes encoding stress proteins such as molecular chaperones responsible for maintaining protein folding [1]. Survival and growth resumption imply successful cellular adaptation to the new conditions as well as the repair of damages incurred to the cell that might compromise its viability. Specific stress conditions elicit distinct cellular responses due to gene expression programs orchestrated by a number of specific transcription factors commonly activated when the cells shift to sub-optimal growth conditions. Among these transcription factors, the basic leucine-zipper (bZIP) proteins form a large multifunctional family, which is conserved in all eukaryotes [2]. These regulators play important roles in the maintenance of cellular homeostasis and in cell differentiation during development in multicellular organisms. They are defined by a basic DNA binding region followed by a leucine zipper motif. In metazoans, bZIP can form hetero-or homodimers, but yeast members of this family mostly act as homodimers [2]. Several subfamilies of bZIP regulators can be defined based on the protein sequences and DNA binding preferences [3]. In this review, we will highlight the role of the Yeast Activator (AP1-like) Protein (Yap) sub-family in the yeast adaptation to environmental stress response. The last section provides an overview of the evolution and functional significance of this family in other fungal species.

## THE YAP FAMILIY OF TRANSCRIPTIONAL REGULATORS

**Figure 1:**
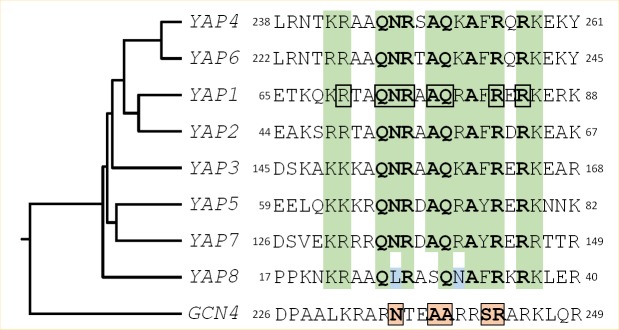
FIGURE 1: Structural features of the Yap family DNA binding domain. The sequences of the eight Yap DNA binding domains (i.e. the basic region of the bZIP motif) are compared with the equivalent region of Gcn4, the classical yeast AP-1 factor, used as an outgroup. A green background highlights the positions, whose physico-chemical properties are conserved in the Yap family. The most conserved residues are in bold. The Yap8 specific residues are in blue. The Yap1 amino-acids which were predicted to contact DNA based on structural studies [12, 140] have been underlined by a black box. The Gcn4 residues involved in DNA interaction are highlighted by pink boxes. The rooted tree and the multiple alignment were obtained from ClustalW (https://www.genome.jp/tools-bin/clustalw), using the bZIP sequences and the 100 flanking amino-acids.

Fifteen bZIP proteins are found in the *S. cerevisiae* genome. Four of them are homologous to the ATF/CREB subfamily (Aca1, Sko1, Hac1 and Cst6) and one is related to AP1 (Jun/Fos) transcription factors (Gcn4). The rest belongs to fungal specific bZIP subtypes [2]. The yeast activator (AP1) protein family is the largest bZIP subfamily in *S. cerevisiae*. It includes eight members (Yap1 to Yap8) which have some sequence similarity to Gcn4. Gcn4 interacts with DNA, via five residues in its basic region (Asn235, Ala238, Ala239, Ser242, and Arg243) that make base-specific contacts with DNA (**Fig. 1**). These residues are highly conserved in the Jun/Fos bZIP proteins found in mammals [4-6]. The Yap family is unusual among bZIP proteins because they contain a glutamine at the position corresponding to Gcn4 Ala239 and a phenylalanine or a tyrosine at the position corresponding to Ser242, hence having different DNA binding properties (**Fig. 1**). Furthermore, there are two family specific residues in the Yap family, in position 234 and 241 of Gcn4 that are a glutamine and an alanine, respectively [7].

Yap1, the first member of the Yap family to be described, was initially identified by its ability to bind a DNA sequence containing the simian virus 40 (SV-40) sequence AP-1 recognition element (ARE: TGACTAA). Based on the ARE-binding capacity, this factor was purified as a 90 kDa protein and the corresponding gene was cloned by screening a λgt11 library with a monoclonal antibody raised against Yap1 [8]. Subsequently, this gene was also found as a multicopy suppressor of sensitivity to the iron chelators 1,10-phenantroline as well as to a variety of drugs, including cycloheximide. Hence, this locus was historically designated as *PAR1*/*SNQ3*/*PDR4* [9]. Besides *YAP1*, a second gene, *YAP2*, conferring resistance to the iron chelator 1,10-phenantroline, was also described. This gene encodes a 45 kDa protein that binds YRE (Yap response elements) located in the promoters of its targets. *YAP2* is also named *CAD1*, due to the acquisition of cadmium resistance in cells overexpressing this gene [10]. The sequencing of *YAP1* and *YAP2* genes revealed the presence of three conserved regions: the bZIP domain in the N-terminus, a region in the C-terminus containing conserved cysteine residues and another one in the internal region adjacent to the bZIP-domain [7].

A search in the *S. cerevisiae* genome using as query the bZIP motif revealed the other six members of the Yap family [11]. All of them possess common key residues in the bZIP, which confer to the family distinct DNA binding properties (**Fig. 1**).

Yap1 recognizes the specific sequences TGACTAA, TTAGTCA, TTACTAA and T(T/G)ACAAA (YREs) in the promoter of its target genes [11-14]. Genome-wide analyses have defined the consensus Yap1 sequence as being TTACTAA (YRE-O) [12, 15, 16]. The remaining Yap transcription factors bind either the YRE-O element (Yap2/Cad1, Yap5, Yap7) or a slightly different motif, TTACGTAA, called YRE-A (Yap4/Cin5, Yap6) [16-19]. Yap3 was described as a transactivator of the YRE-O, but the YRE-A was predicted as his preferred binding motif based on chromatin immuno-precipitation (ChIP-chip) experiments [11-17]. The preference for YRE-O or YRE-A has been proposed to be due to the presence of either an arginine or a lysine in the basic domain of the corresponding Yap (position 15 in the sequences represented in **Fig. 1**) [17], however, this hypothesis is controversial [11, 12]. The sole exception is Yap8/Arr1, which binds a cis-element with 13 base pair sequence TGATTAATAATCA hereafter designated as Yap8 response element (Y8RE) [20, 21]. Both the core element (TTAATAA) and the flanking regions (TGA and TCA) of Y8RE are crucial for Yap8/Arr1 binding and for *in vivo* activation of its targets [20, 21]. Interestingly, a residue in the Yap8 basic region, Leu26, is required for Yap8-DNA binding and Yap8 activity (highlighted in blue in **Fig. 1**). This residue, together with Asn31, hinders Yap1 response element recognition by Yap8, giving its narrow DNA-binding specificity [20].

A structural common feature between *YAP1* and *YAP2* is the presence of unusually long 5'-untranslated region containing short upstream open reading frames (uORF). The *YAP1* leader has one 7-codon uORF whereas the one of *YAP2* contains one 6-codon uORF (uORF1) and an overlapping short reading frame of 23 codons (uORF2), which is located at -1 with respect to the main reading frame [22]. The latter is involved in *YAP2* mRNA turnover via termination-dependent decay. Indeed, the *YAP1*-type uORF allows scanning of 40S subunits to proceed via leaky scanning and re-initiation to the major ORF, whereas the *YAP2*-type acts to block ribosomal scanning by promoting efficient termination [23].

## YAP1, THE REGULATOR OF OXIDATVE STRESS RESPONSE

Cells have to keep intracellular concentrations of peroxides (H_2_O_2_ and organic peroxides) and of other reactive oxygen species (ROS) at very low levels by regulating their concentration through tightly controlled mechanisms. ROS are endogenously produced during aerobic respiration or because of altered cellular environment (oxidant molecules exposure, imbalance in metal homeostasis). Microorganisms, including *S. cerevisiae*, contain sensors that detect the levels of ROS. In yeast cells, Yap1 is activated under oxidative stress conditions and its absence renders cells hypersensitive to the several oxidants that generate superoxide anion radicals [9]. Kuge and Jones provided the first and clear clue towards the role of Yap1 in this response mechanism [24], by showing that *TRX2* gene (thioredoxin) induction by H_2_O_2_, t-BOOH, diamide and diethylmaleate (DEM) is Yap1-dependent. Analyses of several promoter sequences of antioxidants genes such as *TRX2, GSH1* (γ-glutamyl cysteine synthetase) [24], *GSH2* (gluthathione synthetase) [25] and *TRR1* (thioredoxin reductase) [26] revealed functional YREs.

**Figure 2:**
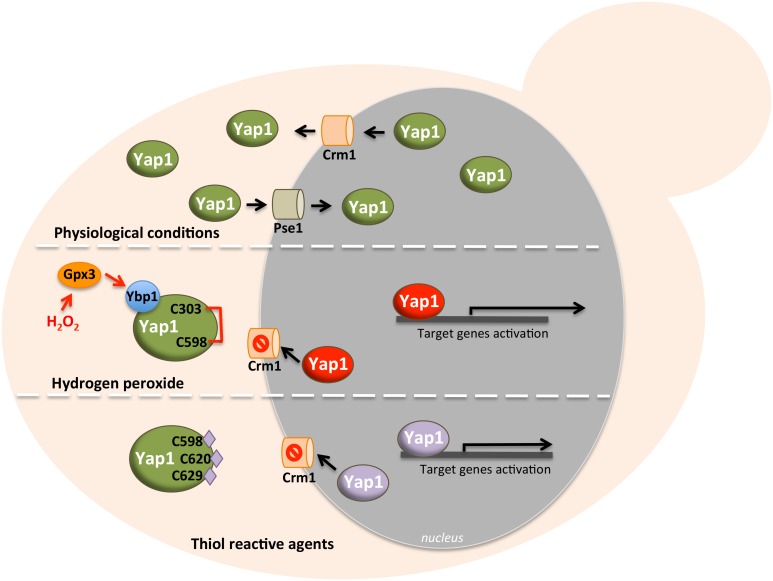
FIGURE 2: Schematic representation of Yap1 activation. Yap1 has two distinct molecular sensors: one for hydrogen peroxide (H_2_O_2_) and the other for thiol-reactive compounds (see description in the text). In the first panel is represented the shuttling of Yap1 between the nucleus and cytoplasm, occurring under physiological conditions, entering the nucleus by Pse1 importin and exiting the nucleus by exportin Crm1. In the second panel is depicted the activation of Yap1 by H_2_O_2_, which is dependent on Hyr1/Gxp3/Orp1 and Ybp1 proteins. H_2_O_2 _induces the formation of a disulfide bond between Cys303 and Cys598 of Yap1, preventing the recognition of the nuclear export signal (NES) by Crm1 (represented in red the activated Yap1). In the third panel is depicted the activation of Yap1 by thiol-reactive agents. These compounds bind to Cys598, Cys620 and Cys629, thereby preventing the recognition of the NES by Crm1 (in purple the activated Yap1). In both cases, the conformational change leads to Yap1 accumulation in the nucleus and posterior gene activation.

Yap1 redox regulation by oxidants involves two cysteine-rich domains (CRDs) located in the N- and C-terminus. Yap1 shuttles between the nucleus and the cytoplasm mediated by the exportin Crm1/Xpo1 and imported by the importin Pse1 [27, 28]. Upon stress conditions, Yap1 accumulates in the nucleus because its NES (nuclear export signal) is masked by the formation of an intramolecular disulfide bond between the cysteine 303 and 598, avoiding Crm1 recognition. Consequently, there is an increased expression of Yap1 targets (**Fig. 2**) [28]. *In vitro* studies performed by Wood M.J. *et al*. revealed that upon H_2_O_2_ exposure, an additional intramolecular disulfide bond between Cys310 in n-CRD and Cys629 in c-CRD is formed [29]. Although this second disulfide bond was not shown to be relevant *in vivo*, it possibly adds stability to the oxidized active conformation of Yap1 [30]. The fact that Yap1 does not respond to H_2_O_2_ in the absence of Hyr1 (also designated as Gpx3 or Orp1), led to establish the role of this protein as a sensor and signal transducer of H_2_O_2_. Yap1 oxidation does not thus take place directly and the Cys36 of Hyr1/Orp1 oxidized to sulfenic acid (Cys36-SOH) senses the H_2_O_2 _signal [31, 32]. Next, the signal is transduced to Yap1 through the generation of an intermolecular disulfide bond between Cys36 of Hyr1/Orp1 and Yap1 Cys598, which then forms the intramolecular disulfide bridge with Yap1 Cys303 rendering Yap1 to its active form (**Fig. 2**) [29]. When this bond is formed, the Cys36 sulfenic acid of Hyr1/Orp1 is prepared to react with its Cys82 to complete the peroxidative cycle. Bersweiler *et al*. showed that the protein Ybp1 could be associated to Yap1 forming a ternary complex with Hyr1/Orp1 [33]. It is possible that Ybp1 functions as chaperoning the formation of the disulfide bridge between the Cys36-SOH of Hyr1/Orp1 with the Cys598 of Yap1. It could also avoid the competition with the Cys36-Cys82 disulfide bond of Hyr1/Orp1 that is part of its catalytic site [33]. The Hyr1/Orp1 peroxidase is different from the classical ones and is reduced by the thioredoxin (Trx) pathway [32, 34].

In contrast to H_2_O_2_, the Yap1 response to diamide is Hyr1/Orp1-independent and it does not involve the n-CRD (**Fig. 2**) [35]. This is consistent with the notion that it possesses two redox centers. Indeed, the electrophile *N*-ethylmaleimide (NEM) and the quinone menadione, both electrophile and superoxide anion generators, were shown to modify the c-CRD cysteines independently of Hyr1/Orp1, leading to Yap1 nuclear accumulation [35]. The Trx pathway is involved in the recycling of the Yap1 oxidized form by disrupting the disulfide bond [36-38]. Fe-S clusters are also very susceptible to oxidation. It was described that Yap1 attenuates the toxic effect of hydroxyurea by regulating the expression of key players of the cytosolic iron–sulfur protein assembly machinery (CIA), proposed to act as sensors of the intracellular oxidative stress [39, 40].

During the oxidation-reduction processes in which Yap1 is active, the glutathione functions either in reduced (GSH) or oxidized (GSSG) form. Recently, it was shown that glutathione in the endoplasmic reticulum (ER) is oxidized but not reduced, being catalyzed to the oxidative state by Ero1, a protein forming the disulfide bond necessary for this process. The reduction of this GSSG molecule to GSH is then occurring in the cytoplasm. As such, the interplay between reduced cytosolic GSH and the oxidized GSSG in the ER keeps the redox homeostasis [41, 42]. The transport of glutathione between the cytoplasm and the ER is facilitated via diffusion through the Sec61 complex (protein-conducting channel) plus the Sec62-Sec63 complex [41, 42].

## YAP1 IN METAL AND METALLOID STRESS

Metal toxicity depends on each metal's physicochemical properties and ligand preferences. Redox-active metals such as iron (Fe), chromium (Cr), copper (Cu) and cobalt (Co) can take oxygen and sulphur as their ligands, whereas redox-inactive metals such as cadmium (Cd) and mercury (Hg) prefer sulphur as a ligand [43-45]. Redox-active metals can induce oxidative stress by participating in Fenton-type reactions, whereas redox-inactive metals imbalance the antioxidant pool of the cell [43-45]. In general, metals induce cellular toxicity by generating oxidative stress, impairing the DNA repair system and inhibiting protein folding and function [45-47].

Yap1 plays a pivotal role in mitigating metal-generated ROS, but its contribution to metal detoxification is not restricted to the induction of the cellular antioxidant defences. Indeed, Yap1 also regulates expression of the *YCF1* gene, the vacuolar transporter of metal-glutathione conjugates, thereby contributing to vacuolar compartmentalization of metals and metalloids such as cadmium and arsenite [48, 49]. Over the last years, several other metal detoxification pathways controlled by Yap1 were unveiled as subsequently detailed.

Cobalt is a biologically relevant metal in many living organisms because it is an essential cofactor of enzymes involved in many reactions [50]. However, when cobalt is in excess it generates oxidative stress, which damages cells. Analysis of transcriptional profiles of cells stressed with high concentrations of cobalt revealed the induction of antioxidant genes in a Yap1 dependent way [51]. Corroborating this molecular data, biochemical analysis showed Yap1 to be important to deal with oxidative damage generated by exposure to cobalt. Activation of Yap1 is not exclusively involved in cobalt-generated ROS since under anoxia, Yap1 also localizes in the nucleus [51]. Moreover, Yap1 up-regulates cobalt uptake through the activation of the expression of the high affinity phosphate transporter *PHO84*, a well-known cobalt transporter. Accordingly, *yap1* knockout cells accumulate lower levels of cobalt [51]. The authors suggested that cobalt accumulation could be a side effect of Yap1 regulation of *PHO84* under non-stressed conditions and proposed that phosphate uptake mediated by Yap1 may fulfill a role in the oxidative stress response triggered by the aerobic metabolism. Reinforcing this possibility, growing evidences indicate that manganesephosphate complexes, which enter cells via Pho84 [52], act as scavengers of superoxide [53, 54].

Cadmium is a well-known mutagenic metal that can enter cells via non-specific divalent metal transporters. Yap1 is a repressor of the *FET4* gene [51], a plasma membrane low affinity iron transporter, which can transport other bivalent metals including cobalt and cadmium ions. Although this repression does not significantly affect cobalt uptake, it avoids cadmium toxicity by impairing its transport into the cell [55]. Genomic deletion of Yap1 increases *FET4* transcripts as well as protein levels [55]. The *yap1* mutant accumulates high cadmium levels compared with the wild-type strain, whilst the deletion of *FET4* gene from the *yap1* mutant resumes cadmium tolerance. Noteworthy, cadmium uptake increased in cells treated with both cadmium and iron because iron induces *CUP1* expression, which possibly binds and sequesters cadmium [55]. Yap1 is not a direct regulator of *FET4* because its promoter does not contain YREs. Previous microarray analysis obtained in the presence of cobalt [51] revealed that Yap1 positively regulates the transcription factor Rox1. This factor is a repressor of hypoxic genes and represses *FET4* expression under aerobic conditions. The promoter of *ROX1* possesses one functional YRE located at – 414 upstream the ATG codon. Yap1 is a direct regulator of *ROX1*, which in turn represses *FET4* [55].

Yap1 also plays an important role in arsenic compound detoxification by regulating genes encoding several of the cellular antioxidant defenses, important to mitigate arsenic-generated ROS [56]. Besides, Yap1 was also shown to control the expression of *YCF1, ACR2*, the yeast arsenate reductase gene, and *ACR3*, the plasma membrane arsenite-efflux protein-encoding gene [48, 57, 58]. Recently, a new line of action of Yap1 in the protection against arsenate toxicity was put forward. By analyzing the transcriptomic profile of Yap1 knockout cells treated with arsenate, several genes involved in the biogenesis of mitochondrial (ISC) and cytosolic (CIA) Fe-S clusters were found to be dependent on Yap1 [59]. This dependence was maintained under anoxia, suggesting that arsenate per se is able to activate Yap1 and triggers the up-regulation of Fe-S cluster biogenesis genes. Arsenate was shown to directly and indirectly (possibly via intracellular ROS production) affect the activity of Fe-S containing proteins and accordingly overexpression of CIA and ISC genes attenuates arsenate deleterious effects [59]. Together these findings led the authors to propose that the transcriptional regulation of Fe-S biogenesis genes may constitute another safeguard against arsenate toxicity activated by Yap1.

## YAP2/CAD1 INVOLVEMENT IN CADMIUM AND OXIDATVE STRESS

Yap2/Cad1 is the family member that shares the highest homology with Yap1 [10]. When overexpressed, Yap2 confers resistance to several stress agents, suggesting a role for this transcription factor in response to toxic compounds. Although *YAP2* and *YAP1* overexpression elicits similar phenotypes, deletion of the latter has strong phenotypic effects, whereas deletion of *YAP2* does not affect or only slightly affects cell growth [7, 10].

Notably, the *YAP2* and *YAP1* single deletion similarly decreased the resistance towards the oxidants H_2_O_2_ and menadione of stationary-phase cultures [60]. Under such circumstances, Yap2 does not regulate the known Yap1 antioxidant targets, an observation that led the authors to propose that the H_2_O_2_-mediated adaptive response could be composed of two distinct regulons, one being controlled by Yap1 and the other by Yap2 [60]. These data also support the notion that Yap1 and Yap2 have overlapping, but not redundant functions. Corroborating this idea, the analysis of the transcriptomic profile of *yap1* and *yap2* null mutants showed that Yap1 and Yap2 activate separated regulons when challenged with H_2_O_2_ [61].

Yap2 transactivation potential is slightly stimulated upon treatment with cadmium [11]. The swapping of Yap1 and Yap2 c-CRDs domains shows that Yap2 cCRD can function in the context of Yap1 in response to cadmium but not in response to H_2_O_2_, indicating the high specificity of these responses [62]. Accordingly, overexpression of *YAP2* in the *yap1* null mutant suppresses cadmium, but not H_2_O_2_ sensitivity [63].

Yap2 is mainly localized in the cytoplasm in unstressed cells but soon after the addition of cadmium it accumulates in the nucleus, activating its targets. In the Yap2 C-terminus, there are three cysteines, Cys391, Cys356 and Cys387, to which cadmium binds directly as shown using the high molecular mass alkylating agent (AMS), which targets free thiol moieties in cysteines. This interaction masks the nuclear export signal recognized by the exportin Crm1 leading to the accumulation of Yap2 in the nucleus [62].

Using proteomic analysis after cadmium treatment, Azevedo *et al*. searched for other Yap2-specific targets [62]. In order to eliminate the influence of Yap1-target genes, a *yap1* null strain transformed with a Yap2 multi-copy vector was used in the presence or absence of cadmium. Proteome analysis under such conditions revealed the induction of the Frm2 protein. The expression of the gene encoding this protein is only dependent on Yap2 in the presence of cadmium. Frm2 shares high identity with type 4 nitrore-ductases, shown to be involved in the fatty acid signalling pathway and required for unsaturated fatty acid control of the stearoyl-CoA desaturase gene (*OLE1*) expression [64]. *FRM2* was also identified in a screen for mutants defective in *OLE1* repression by unsaturated fatty acids, and the fact that the *frm2* mutant is sensitive to arachidonic acid led to the hypothesis that *FRM2* participates in lipid metabolism [62]. Considering that cadmium exerts its toxicity by promoting lipid peroxidation cascades, it is plausible that Yap2 regulates lipid metabolism [65, 66].

Yap2 was also found in a two-hybrid screen using Rck1 or Rck2 MAPK-activated protein kinases (MAPKAPs) as baits [67, 68]. The sensitivity of the *rck1* mutant to tBOOH is fully suppressed by overexpression of Yap2 [67]. Although Yap2 is a cadmium responsive transcription factor, its deletion does not increase yeast sensitivity to cadmium. In the absence of Rck1, Yap2 gives protection against cadmium toxicity. These results indicate that Rck1 appears to have an inhibitory effect on Yap2 activity. Furthermore, Yap2 may play a role in cell wall maintenance by controlling the expression of CWI (Cell wall Integrity) genes, namely *SLT2, RLM1* and *CHS1* [68, 69]. These genes are dependent on Yap2 but not on Yap1 and in the *yap2* mutant strains, CWI genes are downregulated in the presence of cadmium indicating a regulatory role of Yap2 on their expression. It is possible that cadmium causes damage in the glucan structure of the cell wall, thus activating the expression of the CWI genes [68, 69].

## YAP3, A TRANSCRIPTION FACTOR WITH A POTENTIAL FUNCTION UNDER HYDROQUINONE STRESS

Yap3 (YHL009C) encodes a 399 amino acid protein containing a bZip domain similar to the other family members (**Fig. 1**). *YAP3* is located in the chromosome VIII and activates transcription from promoters containing a Yap recognition element (YRE; 5'-TTAC/GTAA-3') [11]. *YAP3* is not an essential gene and so far, the regulatory targets of Yap3 are not yet defined but it seems that Yap3 plays a specific role in the cellular response to hydroquinone (HQ). Indeed, the *yap3* mutant strain is sensitive to HQ. Like other Yap family members, Yap3 contains two CRDs and a NES in its C-terminus. Yap3 localizes in the nucleus upon treatment with HQ [70]. Yap3 also responds to ER stress, as the null mutants are sensitive to tunicamycin, a compound that causes ER stress through induction of the unfolded protein response [71]. Yap3 was also identified in a screen of wild-type and mutant strains as being sensitive to arsenic (As) and monomethylarsonous (MMA) treatments, suggesting that these stresses and HQ share cellular targets [72].

Yap3 possesses a very high transactivation potential, even higher than the one of Yap1 in the absence of any stress [11], suggesting that this transcription factor might have an important function which was not yet precisely identified.

## YAP5 CONTRIBUTES TO IRON HOMEOSTASIS

Yap5 (YIR018W) is a protein containing a CRD at the C-terminus and a bZIP domain at the N-terminus that recognizes YRE-O sites (**Fig. 1**). Additionally, Yap5 possesses a Hap4L domain just upstream of its bZIP. The Hap4L motif is a conserved protein sequence of 16 amino acids, which is found in proteins interacting with the CCAAT binding complex (CBC), a highly conserved transcriptional regulator [73]. However, the Hap4L domain of Yap5 is degenerated compared to other CBC interacting proteins and its role in Yap5 activity in *S. cerevisiae* has not been investigated yet [19]. Yap5 is responsible for yeast adaptation to iron overloading conditions. The Ccc1 transporter fulfills an important role in high iron detoxification, by importing iron into the vacuole, which is the major site of iron storage in fungi and plants [18]. *CCC1* expression is induced by iron through the activity of Yap5 [18, 74]. However, the up-regulation of *CCC1* expression driven by Yap5 is not essential for cells to cope with high iron toxicity, since deletion of the functional YRE [18] from the *CCC1* promoter region still allows cell growth under high iron levels [74]. Corroborating this notion, the *yap5* null mutant is not as sensitive to iron excess as the *ccc1* null mutant is [74-76].

**Figure 3:**
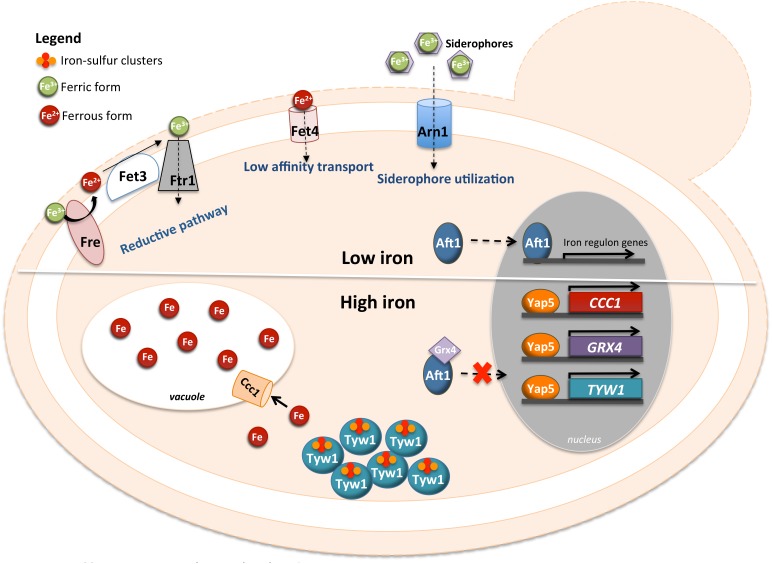
FIGURE 3: Schematic representation of the Yap5 involvement in the cellular response to iron-overload. In upper panel is represented the cellular response to low levels of iron (“low iron” in the figure) where the Aft1 transcription factor is responsible for the activation of the iron regulon. In the lower panel is represented the role of Yap5 when cells are exposed to iron excess conditions (“high iron” in the figure). Yap5 activates the expression of *CCC1*, coding for a vacuolar iron transporter, and of *TYW1*, encoding a cytosolic Fe-S cluster protein. Additionally, it activates the expression of *GRX4* gene, coding for a glutathione-dependent oxidoreductase, leading to Aft1 accumulation in the cytoplasm.

Transcriptional and chromatin immunoprecipitation analyses revealed two other genes directly regulated by Yap5 with a relation to iron homeostasis, *TYW1* and *GRX4* [74, 77, 78]. *TYW1* encodes a cytosolic iron-sulfur (Fe-S) cluster-containing enzyme required for the synthesis of Wybutosine modified tRNA [79]. It was proposed that the induction of *TYW1* triggered by Yap5 might provide protection against iron toxicity by sequestering cytosolic free iron as protein-bound Fe-S clusters [77]. *GRX4* encodes a cytosolic monothiol glutaredoxin, which together with Grx3 inhibits Aft1 activity under iron loading conditions by promoting its retention in the cytoplasm [80, 81]. The Yap5-dependent up-regulation of *GRX4* expression was suggested to reinforce this function, as in the *yap5* null mutant Aft1 nuclear exclusion is slightly impaired [74] (**Fig. 3**).

While the transcriptional activity of Yap5 depends on iron bioavailability, Yap5 binding to the promoter of its target genes is iron insensitive [18, 74] and Yap5 is constitutively localized in the nucleus. Iron sensing by this transcription factor depends on two CRDs (amino- and carboxyl-terminal CRDs), located in the C-terminus of the protein and separated by 37 residues [18]. Mutations of the cysteine residues of the CRDs impair the induction of Yap5 targets and compromise the adaptation of yeast to high iron [18].

Yap5 activation by iron is abrogated by mutations in genes encoding proteins of the mitochondrial iron-sulfur cluster assembly system (ISC), indicating that the transcriptional response to high iron is dependent on Fe-S biogenesis [82]. Accordingly, Rietzschel *et al*. showed that Yap5 senses iron by coordination of Fe-S clusters [76]. Both CRDs of Yap5 bind a 2Fe-2S cluster, whose maturation is uncommon as it depends on the ISC but not on the cytosolic CIA machinery [76]. These authors found that Fe-S cluster binding to Yap5 induces a conformational change in the protein, which may explain the increase in its transactivation potential under conditions of iron excess.

## THE ROLE OF YAP4/CIN5 AND YAP6 DURING OSMOTIC STRESS

Hyperosmotic stress leads to the passive efflux of water from the cell to the exterior, resulting in a decrease in cell volume, loss of the state of turgidity, resulting in rigidity and increased concentration of cellular solutes [83]. In the case of hypo-osmotic environment, it allows the movement of water into the cell, originating the swelling of the cell, a high-pressure turgor as well as the dilution of the intracellular milieu [83]. To counteract these effects, the cell makes use of osmolytes, which are compatible solutes, such as the alcohol glycerol, trehalose, and sorbitol that protect the cell against the effects of an osmotic challenge by modifying the intracellular osmotic pressure [83-85]. These alterations correlate to modifications of gene expression that consequently leads to the alteration of the cell permeability to the osmolytes and of their biogenesis rate. These metabolic alterations are triggered by the HOG (high osmolarity glycerol) pathway via the modulation of the expression of stress-responsive genes [86]. The main actor of this pathway is Hog1, a mitogen-activated protein kinase (MAPK). Hog1 controls the activity of several transcription factors, in particular Msn1 [87], Msn2/4 [88], Hot1p [88], and Sko1p [86], among others.

Yap4/Cin5, the fourth member of the Yap family was initially characterized as a chromosome instability mutant designated as *Cin5*, encoding a 33 kDa protein [89]. Its overexpression confers salt tolerance [90] as well as resistance to antimalarial drugs [91] and cisplatin [92]. Results from several microarray analyses indicate an induction of Yap4 under various conditions, such as those of oxidative and osmotic stress. The *YAP4* null mutant reveals a slight salt-sensitivity phenotype under hyperosmotic stress. On the other hand, it was also shown that under these conditions *YAP4* gene expression is regulated by Msn2 through the more proximal STRE elements (-430bp). The promoter of *YAP4* contains several *cis*-elements such as HSE (nGAAnnTTCn) located at -432 and -425 upstream of ATG codon and bound to HSF, a Yap1-cis element located at -516 and -508 from the ATG codon (YRE – TTAG/CTAA) and also STRE *cis*-elements for Msn2/Msn4 (AGGGG) [90]. Moreover, Yap4 is a downstream component of the HOG pathway and its overexpression partially rescues the salt sensitive phenotype of the *hog1* single mutant [90].

Yap6 is a 44 kDa protein sharing almost 20% identity with Yap4/Cin5, making it the closest-related Yap family members (**Fig. 1**). Overexpression studies in the *ena1* mutant (lacking the Na^+^/Li^+^ extrusion ATPase) subsequently identified both *YAP4/CIN5 (HAL6)* and *YAP6 (HAL7*) as genes that confer salt tolerance through a mechanism unrelated to the Na^+^/Li^+ ^ATPase extrusion [93]. In contrast to Yap1, Yap2 and Yap8, the subcellular localization of Yap4 and Yap6 is constitutively nuclear.

Yap4 also interacts with the product of the yeast gene *LOT6* (YLR011W) encoding a 21 kDa protein. Lot6 possesses a quinone reductase activity similar to its mammalian counterparts [90, 94, 95]. The association of Yap4 with this quinone reductase and the 20S proteasome affects its ubiquitin-independent degradation. It was proposed that the FMN cofactor in the Lot6 active site is a redox-regulated switch that controls the stability and localization of Yap4 [96]. A similar redox-controlled mechanism might regulate p53 and related transcription factors in mammalian cells [97]. It was also reported that the association of Lot6 with the 20S proteasome is via its flavin-binding site [96]. Interestingly, however, these authors showed that the reduction of the FMN cofactor by either NADH or light irradiation results in the binding of Yap4 to the Lot6–proteasome complex, indicating that recruitment of Yap4 depends on the redox state of the quinone reductase [96]. Alternatively, Lot6 in its native dimeric state is essential for the binding of Yap4 to the complex. The dissociation of Lot6 dimers into monomers does not affect the catalytic properties of the enzyme with regard to quinone reduction [98]. These authors put forward the hypothesis that Yap4 binds the Lot6:20S proteasome in a redox-dependent manner and may participate in the proapoptotic effect of Lot6 and thus might represent an activator of yeast apoptosis [98].

Transcriptional arrays of the *yap4* mutant under mild conditions of hyperosmolarity revealed a large set of genes possibly regulated by Yap4. Amongst these target genes are *GCY1*, encoding a putative glycerol dehydrogenase, and *GPP2*, encoding a NAD-dependent glycerol-3-phosphate phosphatase. These genes show a decrease of their induction in the *yap4* mutant strain with reduction values corresponding to 40% and 50% of the maximum levels, respectively. Furthermore, *DCS2*, a gene homologous to the *DCS1*-encoded mRNA decapping enzyme, shows 80% reduction of its induction level in the *yap4* mutant upon osmotic shock. The fact that *YAP4* and *YAP6* are induced by a variety of unrelated forms of environmental stresses suggests a universal role in the yeast response to stress, in contrast to the other Yap members [90].

ChIP-chip experiments have shown that Yap4/Cin5, Sko1, Yap6, Msn2 and Skn7 bind their targets after incubation with high salt (0.6 M) for 30 min [99, 100]. Later, Ni *et al*. determined that the binding of several of these transcription factors is a dynamic process [101]. Their data allowed the classification of Yap4 targets into three classes: constant binding independently of salt (class 1), rapid induction (class 2) and slow induction (class 3). Other minor binding patterns were found such as transient induction (class 4) and decrease in binding (class 5). Sko1 and Yap6 also bound many Yap4 constitutive targets of class 1, at either 0 min or 30 min. Another interesting aspect is that Msn2 preferentially binds inducible Yap4 targets. Moreover, Yap6 and Sko1 bind a significant number of salt-induced Yap4 targets that belong to class 2. It seems from the results of Ni *et al*. that the binding of other factors correlates with induced binding, and thus the association of different components at induced targets regulates gene expression [101]. Yap4 targets were involved in oxidoreductase activity and Yap4, together with Sko1, have targets involved in hexose transport, glucose and ethanol catabolism. Yap6 has targets in the same categories as Yap4 but it specifically targets genes encoding ribosomal proteins.

Yap4 is a highly phosphorylated protein. This post-translation modification is dependent on PKA and GSK3 and was shown to affect its stability but not its nuclear localization [102].

Finally, Yap4 and Yap6 were shown to interact with the general transcriptional repressor Tup1, suggesting that they could also act as transcriptional repressors [16, 93].

## YAP7 AND NITROSATIVE STRESS

The function of Yap7 *(YOL028c)* has not been completely deciphered. It was described that Yap7 represses *YHB1*, encoding a flavohemoglobin which functions as a nitric oxide (NO) oxidoreductase [19]. In consequence, Yap7 deletion confers high resistance to NO. Yap7 repression of *YHB1* is exerted by binding YRE-O motifs in the *YHB1* promoter and by recruiting the transcriptional repressor Tup1 [19, 103]. Like Yap5, Yap7 has a bipartite Hap4L-bZIP domain, which was shown to play a role in its function. However, the de-repression of *YHB1* observed in a mutant of CBC is only 30% of that observed in a *yap7* mutant, indicating that Yap7 repressor activity is only partially dependent on CBC [19]. Noteworthy, in laboratory *S. cerevisiae* strains, *YAP7* is interrupted by a frame-shift and produces a truncated protein which has DNA binding properties but lacks the Tup1 interaction domain and is unable to repress transcription. The role of Yap7 was therefore revealed by studying “wild” yeast strains expressing a full-length Yap7 protein [19].

## YAP8 AND ITS ROLE IN THE DETOXIFICATION OF ARSENIC COMPOUNDS

Arsenic (As) is the 20^th^ most abundant element in the earth's crust and is a highly toxic metalloid with respect to the human health being the most potent human carcinogen. Although synonymous to a poison, it is one of the oldest drugs in the history of humankind, first used to treat syphilis and later malaria. In spite of its toxic effects, arsenic is also a chemotherapeutic agent in the treatment of the acute promyelocytic leukemia (APL) as well as other solid cancers [104-108]. Arsenic is a multifactorial element because this metalloid interferes with several metabolic pathways leading to a myriad of cytotoxic effects, forming ROS with the induction of apoptosis [56].

**Figure 4:**
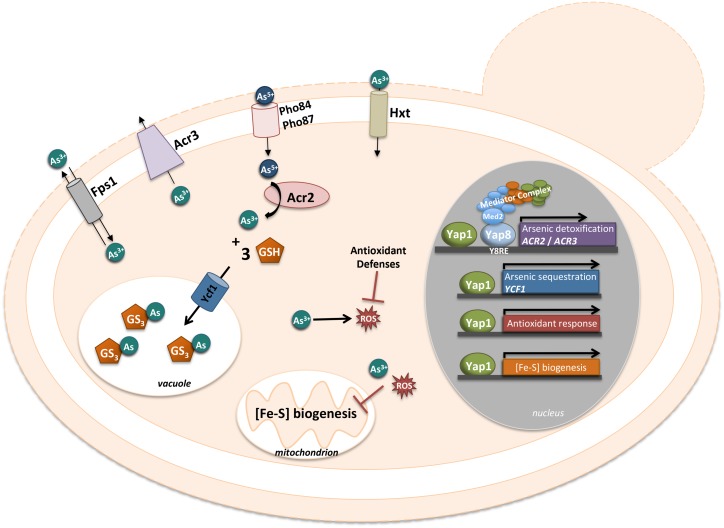
FIGURE 4: Schematic representation of the Yap1 and Yap8 involvement in arsenic adaptation. The phosphate transporters, Pho84 and Pho87, take up arsenate. Arsenite can enter the cells through hexose transporters, Hxt, and the aquaglyceroporin, Fps1. Upon arsenic exposure, Yap8 recognizes and binds a specific YRE sequence, TGATTAATAATCA, depicted as Y8RE. Then, it interacts with the mediator complex, via the tail subunit Med2, which is essential for the full activation of its target genes, *ACR2* and *ACR3* (for details see text). Arsenite is imported into the vacuole, in conjugation with glutathione, by Ycf1, which is regulated by Yap1. Furthermore, Yap1 activates antioxidant response and Fe-S cluster biogenesis genes, to mitigate the ROS and the disruption of Fe-S clusters, generated by arsenic.

The global environmental widespread of arsenic led the organisms to develop and evolve several detoxification mechanisms for this compound. In *S. cerevisiae* the main detoxification system for arsenic is composed of Acr2/Arr2, an arsenate reductase responsible for the reduction of arsenate (As(V)) to arsenite (As(III)) [109] and Acr3/Arr3, a dual As(III) and antimonite (Sb(III)) plasma-membrane efflux protein [45, 110]. The transcription factor Yap8/Arr1 is the master regulator of arsenic detoxification that binds the promoter region located between *ACR2* and *ACR3*, two divergently transcribed genes (**Fig. 4**). Remarkably, the *YAP8* gene itself is located just next to *ACR2* and *ACR3*, hence forming a genomic cluster specialized in arsenic resistance. Yap8 specifically recognizes and binds an extended YRE with a 13 bp pseudo-palindromic sequence (TGATTAATAATCA), where both the core element (TTAATAA) and the flanking sequences are essential for Yap8 binding and transcriptional activation of its targets (**Fig. 4**) [20, 45]. Yap8 was found to be regulated by arsenic at the level of its nucleo-cytoplasmic shuttling. When exposed to arsenic the cysteine residues, Cys132, Cys137 and Cys274, bind the arsenic compound masking the NES and as such, Yap8 will remain in the nucleus activating its targets [57]. However, contrary to these observations, another study showed that Yap8 is constitutively nuclear, being associated with the *ACR3* promoter in untreated as well as As(III)-exposed cells [45]. Unknown genetic differences in *S. cerevisiae* strains used and/or different expression systems may account for the discrepancies between both works. The three conserved cysteine residues, Cys132, Cys137 and Cys274, are important for Yap8 transactivation function, since the mutants obtained by substitution for each residue failed to induce *ACR3* expression [57]. Menezes *et al*. have shown that arsenate alters the sulfhydryl state of Yap8 conserved cysteines, suggesting that these residues may also be direct sensors of the pentavalent form of arsenic, As (V) [111].

As(V) also induced the expression of the Aft1-dependent gene *CTH2* to levels similar to those triggered by BPS (bactophenantroline disulfonic acid) [112]. However, in the presence of As, the expression of the high-affinity iron uptake protein encoding genes *FET3* and *FTR1* is abrogated and the *aft1* mutant growth is impaired. Furthermore, growth of the *aft1* mutant in presence of As is totally resumed when iron is added, indicating a physiological link between As and the Aft1/2 regulon iron deprivation response [112]. As such, these data revealed that As(V) causes Fe scarcity.

Tamas's laboratory showed that Yap8 escapes degradation under arsenic conditions [113]. Later, Ferreira *et al*. have shown how Yap8 circumvents proteolysis under As stress [114]. Although Ufd2, an E4-Ubiquitin ligase, was involved in protein degradation, those authors found that the *UFD2* deletion causes Yap8 degradation and a decrease in its transcriptional activity. Consequently, the cell growth under arsenic stress is compromised in this mutant. These data suggested that Ufd2 possesses another function besides proteolysis. Several reports indicated that the Ufd2 U-box motif is essential for ubiquitination and as such, is required for Ufd2 action during proteolysis [115, 116]. However, the U box motif is not functional in the Ufd2 that is acting as a stabilizing protein in the activity of Yap8 [114].

Another important aspect of Yap8 activity relies on its interaction with the core transcriptional machinery and more particularly with the Mediator complex (**Fig. 4**). The Mediator is a complex molecular machine composed of about 20 subunits organized in four domains (tail, middle, head and regulatory modules) [117-119]. The tail module contains the subunits Med2, Med3, Med15 and Med16 interacting with transactivators and as such recruiting the complex to the gene promoter. Until now a small numbers of associations between the Mediator tail subunits and transcription factors such as Pdr1/Pdr3 [120], Hsf1 [121], Gal4 [122], and Gcn4 [123] were described. Using two hybrid assays in the presence of arsenate, Menezes *et al*. revealed that Yap8 is a partner of Med2, a result afterwards confirmed by chromatin immunoprecipitation assays [111]. After Yap8 activation, the Mediator binds the *ACR2*/*ACR3* promoter through the interaction with the mediator complex via the tail subunit, Med2 (**Fig. 4**). Transcription is also under the control of the SWI/SNF and SAGA chromatin-remodeling complexes [124-126]. In the case of Yap8, the specific SWI/SNF and SAGA subunits (Snf2, Snf5 and Spt20) are as well required for the full expression of *ACR2*. In conclusion, Menezes *et al*. showed that Yap8 is a direct sensor of arsenate and that the Mediator and chromatin-remodelers SWI/SNF and SAGA are essential coactivators for the expression of Yap8 targets, *ACR2* and *ACR3* [111].

## THE YAP FAMILIY IN OTHER FUNGAL SPECIAS: AN EVOLUTIONARY PERSPECTIVE

**Figure 5:**
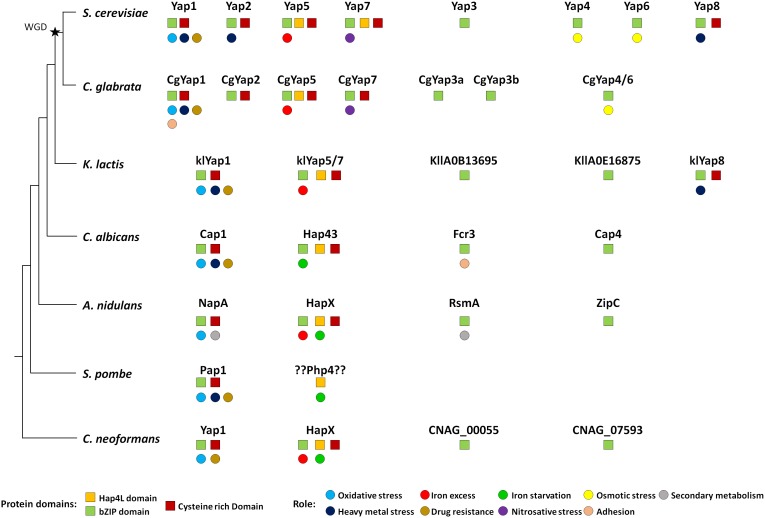
FIGURE 5: Evolution of the Yap family in fungi. The Yap proteins have been indicated for seven fungal species: the hemiascomycetes *S. cerevisiae, C. glabrata, K. lactis* and *C. albicans*, the euascomycete *A. nudilans*, the archaeascomycetes *S. pombe* and the basidiomycete *C. neoformans*. The tree on the left is just a schematic representation of the phylogenetic relationships between these species. The whole genome duplication event is indicated by a black star. The remarkable protein domains (squares) and the described function (circles) are indicated for each Yap. The color code is indicated at the bottom of the figure.

Orthologues of Yap transcription factors are found in all fungi. Most species have three to four members of this family, with the notable exception of the fission yeast *Schizosaccharomyces pombe*, which has only one (**Fig. 5**). The eight *YAP* genes described in *S. cerevisiae* actually originated from whole genome duplication (WGD), which occurred in its ancestry. This WGD created pairs of ohnologues (*i.e*. paralogues arising from the WGD). It was followed by massive gene reduction and most of the ohnologues were lost. Consequently, the modern yeast species which have encountered the WGD (named post-WGD species) have roughly the same number of genes than those which haven't (called pre-WGD species) [127]. Yet, in some cases, the ohnologues evolved divergent functions which were positively selected and retained. This was the case for the Yap family and most extent post-WGD yeast species have six to eight *YAP* genes, with some variations in the repertoire. In *S. cerevisiae*, three pairs of ohnologues were retained: Yap1/Yap2, Yap4/Yap6 and Yap5/Yap7 (**Fig. 1**). Another well-studied post-WGD species, the human pathogen *Candida glabrata*, has seven *YAP* genes: it lacks an orthologue for *YAP8*, has two *YAP3* genes (named *CgYAP3a* and *CgYAP3b*) and only one orthologue for the *YAP4* and *YAP6* pair (named *CgYAP4/6)* (**Fig. 5**) [103].

Yap1 is the most conserved member of the family, both in terms of sequence and in terms of function (**Fig. 5**). Orthologues playing a major role in oxidative stress response have been described in post-WGD yeast species (e.g. *Cg*Yap1 in *C. glabrata*), pre-WGD yeast species (e.g. *Kl*Yap1 in *Kluyveromyces lactis*, Cap1 in the human pathogen *Candida albicans*), euascomycetes (e.g. NapA in *Aspergillus nidulans*), archeascomycetes (e.g. Pap1 in *S. pombe*) and basidyomycetes (e.g. Yap1 in the human pathogen *Cryptococcus neoformans*) [128-134]. Although their list of target genes diverged, they all share a core set of regulated genes which are essential for redox homeostasis (e.g. catalase, thioredoxin, thioredoxin dependent peroxydases, glutathione reductase, etc.) [12, 17, 135-138]. All the Yap1 orthologues recognize YRE-O motifs [12, 139], but *Cg*Yap1 and Pap1 are also able to interact with YRE-A sites [17, 140]. The mechanism for Yap1 redox sensing is remarkably conserved. In *C. glabrata*, the overexpression of Ybp1 confers resistance to oxidative stress and *Cg*Yap1 cooperates with *Cg*Skn7 to regulate a set of protective genes under oxidative stress, as described in *S. cerevisiae* [136, 141]. In *C. albicans*, Cap1 is regulated at the level of its nucleocytoplasmic localization due to the interaction of its CRDs with Ybp1 and Hyr1/Orp1/Gpx3, as described for Yap1 in *S. cerevisiae* [142, 143]. Notably, Cap1 mutants are defective for macrophage escape and less virulent than wild-type strains [142]. In *S. pombe*, Crm1 actively exports Pap1 from the nucleus [134, 144, 145]. Pap1 is activated by the oxidation of its CRDs by the peroxiredoxin Tpx1 [146-149].

The involvement of Yap1 in multidrug resistance is also largely conserved (**Fig. 5**). Null mutants for *YAP1* orthologues show sensitivity to multiple drugs in *C. glabrata, K. lactis, C. albicans, S. pombe* and *C. neoformans* [131, 133, 150-153]. The role of Yap1 and Yap2 in cadmium detoxification is slightly less widespread. Cadmium sensitivity has been observed for mutants of Yap1 orthologues in *C. glabrata, K. lactis, C. albicans, N. crassa* and *S. pombe*, but not in *A. nidulans* and *C. neoformans* [103, 129-131, 133, 134, 150, 154]. Besides these conserved roles, some Yap1 orthologues have more species-specific functions. For instance, in *C. glabrata, Cg*Yap1 controls the expression of the specific adhesin Epa2, which is involved in host colonization [155]. In *A. nidulans*, NapA modulates secondary metabolites production and sexual development [156-158].

The Yap5 and Yap7 proteins show poor sequence conservation over the full sequence. Still, their lineage can be easily traced over large evolutionary distances due to their characteristic Hap4L-bZip bipartite domain, which provides them with the potential to interact both with the CCAAT binding complex and with DNA [73, 159-161]. Moreover, most of them have retained the specific CRD, which is used by Yap5 to sense iron-sulfur clusters [76, 162]. Orthologues have been found in *C. glabrata* (*Cg*Yap5 and *Cg*Yap7), *K. lactis* (*Kl*Yap5/7), *C. albicans* (Hap43) and in euascomycetes and basidyomycetes (HapX). These proteins are consistently involved in fungal iron homeostasis and their interaction with the CBC is conserved in most species and required for their function (**Fig. 5**) [18, 19, 159, 160, 163, 164]. As for Yap1, a core set of targets involved in iron consuming is remarkably conserved between Yap5, *Cg*Yap5, Hap43 and HapX [74, 103, 164-166]. However, in contrast to the Yap1 case, their precise role has been considerably rewired during evolution. In euascomycetes, HapX is involved in both iron starvation and iron excess responses, by repressing or activating the expression of the iron consuming genes depending on the iron supply [162]. In basidyomycetes, HapX is also able to act both as a repressor and as an activator [164]. In the pre-WGD yeast *C. albicans*, Hap43 is mostly involved in the repression of iron consuming genes when iron is limiting and it has almost no role in the iron excess response [160, 163, 167]. Conversely, in *C. glabrata* and *S. cerevisiae* Yap5 is majorly involved in the iron excess response and has few impacts on the iron starvation response [18, 19, 73, 74, 103]. This role of Yap5 in the protection against iron overload is conserved in the pre-WGD yeast species *K. lactis* and *Lachancea kluyverii* [19].

No Hap4L-bZIP bipartite protein is found in *S. pombe*. However, a functional homologue of HapX, named Php4, has been described in this species. Php4 has a Hap4L domain but no bZIP. It represses the expression of iron consuming genes under iron limited conditions through its interaction with CBC, but it has no role in iron excess response and does not bind DNA directly [168, 169]. In contrast to HapX and Yap5, Php4 senses iron indirectly through interaction with glutaredoxins [170]. Then, it is difficult to say if Php4 is actually a HapX orthologue, which has lost its bZip domain, or if the similar role of Php4 and HapX in iron starvation response is just an evolutionary convergence between proteins of different origins.

The role of Yap7, the ohnologue of Yap5 in the constitutive repression of *YHB1* is conserved in post-WGD species but not in the pre-WGD species *K. lactis* and *L. kluyverii* [19]. This led to propose that this role appeared after the WGD. However, this hypothesis is challenged by the fact that *YHB1* is a target of Hap43 in *C. albicans*, which represses its expression in a Tup1-dependent way when iron is limiting [163]. Notably, *YHB1* is a heme-containing protein and therefore the activity of Yap7 is also connected to iron homeostasis. Consequently, the deletion of *YAP7* confers resistance to iron overload in *C. glabrata*, probably due to the high expression of Yhb1 which traps iron into a protein-bound, non-toxic, form [103]. Importantly, HapX proteins are required for the pathogenesis of several fungal pathogens of human and plants [163, 166, 171, 172], but they are dispensable for virulence in *C. neoformans* and in the dermatophyte *Arthroderma benhamiae* [164, 173].

The mode of action and DNA interaction properties of this sub-group of Yap proteins has also diverged. In euascomicetes and in *C. albicans*, the interaction with CBC is predominant, the most highly enriched motif in the promoter of the targets of HapX, and Hap43 is the CCAAT motif [162, 165]. Still their bZIP domain is important for their regulatory properties, but it only synergically contributes to DNA binding with a loose specificity [160, 174]. In *C. glabrata* and *S. cerevisiae*, the YRE-O is the most enriched motif in the target promoters of Yap5 and it is necessary for the binding to occur [18, 77, 103]. In *C. glabrata*, the interaction with the CBC is also necessary for the activity of Yap5 on its targets and a CCAAT motif is always found close to the YRE in the promoter of its targets [73]. This aspect of Yap5 regulation has not been investigated in *S. cerevisiae* yet. In post-WGD species, the Yap7 lineage is apparently on the way of losing the CBC interaction. Indeed, the CBC only partly contributes to the repression properties of Yap7 in *S. cerevisiae* [19]. In *C. glabrata, Cg*Yap7 has even totally lost its Hap4L domain and does not require CBC for its function [19].

In terms of sequence, Yap3 is the second most conserved Yap after Yap1 and *YAP3* orthologues can be found with good confidence from *S. cerevisiae* to euascomycetes (**Fig. 5**). Yet, in most species, no clear role could be assigned to these regulators. In *C. glabrata*, large-scale analyses failed to identify a biologically meaningful set of targets for Yap3a and Yap3b [103]. In *C. albicans, FCR3* was initially described as a partial multicopy suppressor for the fluconazole sensitivity of a *pdr1*Δ*pdr3*Δ *S. cerevisiae* strain. However, Fcr3 has no role in drug resistance in *C. albicans*. The only phenotype described for *FCR3* null mutants is a decrease in adherence properties [175]. In *A. nidulans* and in the human pathogen *Aspergillus fumigatus*, RsmA stimulates secondary metabolite production and controls sexual development [158, 176-178]. However, these processes are specific for filamentous fungi and cannot be transposed to yeasts.

The Yap4 and Yap6 lineage shows high sequence divergence. Still probable orthologues can be identified in almost all fungal species (**Fig. 5**). It is not clear if the role described for these two factors in the osmotic stress response of *S. cerevisiae* is conserved in other species. In *C. glabrata*, about 40 targets of *Cg*Yap4/6 have been identified, with no obvious connection with osmotic homeostasis. Yet, *CgYAP4/6* null mutant exhibits a moderate growth defect in high salt concentration conditions [103]. The orthologues of Yap4 and Yap6 in *C. albicans* (Cap4) and *A. nidulans* (ZipC) have no functional annotation, but their potential involvement in the osmotic shock response has not been investigated to our knowledge.

Yap8 shows a strange and hectic conservation pattern. Orthologues are found in *S. cerevisiae* and two closely *Saccharomyces sensu stricto* species, but in no other post-WGD yeasts. Additionally, *YAP8* orthologues are present in a handful of pre-WGD species, namely *K. lactis*, two *Lachancea* species (out of twelve which genomes is fully sequenced) and *Torulaspora microellipsoides* (information taken from www.saccharomycessensustricto.org and from gryc.inra.fr). Each time *YAP8* is present in a genome, an *ACR3* orthologue is found just next to it on the same chromosome, hence constituting a small genomic cluster involved in arsenic resistance. Intriguingly, although the presence of *YAP8* is poorly conserved in yeasts, the sequence identity between the Yap8 orthologues is high. For instance, the *S. cerevisiae* and *L. fermentati* Yap8 proteins share 46% identity over the full sequence, despite of the large evolutionary distance separating these two species. For comparison, the *S. cerevisiae* and *L. fermentati* Yap1 proteins share only 33% identity. Is it a sign of an introgression of the *YAP8* locus from one species to the other? Or has Yap8 been conserved in this particular species because of special environmental selective pressures? The current knowledge does not allow answering these questions. Importantly, the role of Yap8 in arsenite resistance and its property of direct sensing of As(III) molecules are conserved in *K. lactis* [179, 180].

## CONCLUSIONS

Data obtained in the last decade have shown that gene expression regulation under stress conditions does not involve a single transcription factor but cooperation between several such factors. For instance, *RPN4*, which encodes a transcriptional activator of proteasome genes, contains in its promoter multiple regulatory elements bound by Hsf1, Pdr1/Pdr3 and Yap1 [181] and Yap4/Cin5 contains also in its promoter HSE/Hsf1, STRE/Msn2/Msn4 and YRE/Yap1 elements [90]. Then, responses to stress are not linear sequences of events but rather an orchestrated phenomenon that puts at play several interconnected pathways and response elements, acting via condition and gene-specific cross-talk events. This would lead to a precise response and adaptation to the new environment. In this review, we have thus focused on the major transcription factors of the Yap family that are involved in yeast stress response. It will be important to understand how the activity of these factors is coordinated, as well as to identify the signals triggering this coordination and to determine their integration with metabolic pathways. The work by Snyder's group [101] shows the interaction of factors such as Yap4/Cin5, Yap6, Sko1, Msn2 and Msn4. Furthermore, it would be attractive to study how these transcription factors interact with each other. It could be directly or indirectly via the transcriptional machinery as it was already determined for Yap8 [111]. Another important point is that several Yap transcription factors (Yap4/Cin5, Yap5, Yap6, HapX) can act as both inducers and repressors. The precise mechanisms behind this versatile activity might constitute another line of research once all the targets of these transcription factors have been known.

## Abbreviations

ARE: AP1-recogniction element
bZIP: basic leucine-zipper
CBC: CCAAT binding complex
CIA: cytosolic iron–sulfur protein assembly
CRD: cysteine-rich domain
CWI: cell wall integrity
ER: endoplasmic reticulum
GSH: reduced glutathione
GSSG: oxidized glutathione
HOG: high osmolarity glycerol
HQ: hydroquinone
NES: nuclear export signal
NO: nitric oxide
ORF: open reading frame
ROS: reactive oxygen species
Trx: thioredoxin
uORF: upstream ORF
WGD: whole genome duplication
Y8RE: Yap8 response element
YRE: Yap response elements
